# Does Collective Interest or Self-Interest Motivate Mask Usage as a Preventive Measure Against COVID-19?

**DOI:** 10.1017/S0008423920000475

**Published:** 2020-05-14

**Authors:** Clifton van der Linden, Justin Savoie

**Affiliations:** 1 Department of Political Science, McMaster University, 1280 Main Street West, Hamilton, Ontario L8S 4M4; 2 Department of Political Science, University of Toronto, 100 St. George Street, Toronto, Ontario M5S 3G3

## Abstract

The revised guidance on masks from public health officials has been one of the most significant COVID-19 policy reversals to date. Statements made at the outset of the pandemic, including those from the World Health Organization (WHO), the United States Surgeon General, and the Chief Public Health Officer of Canada, all actively discouraged asymptomatic members of the general public from wearing masks. However, on April 3, 2020, the United States Center for Disease Control and Prevention (CDC) issued new recommendations that called for nonmedical masks, such as cloth face coverings, to be worn in public settings where other social distancing measures are difficult to maintain (Adams, 2020). Canadian public health officials quickly followed with their own guidance for wearing nonmedical masks or face coverings when out in public; however, they have stressed that doing so is optional for asymptomatic persons and should be seen as a complement to existing precautionary measures such as physical distancing and hand hygiene, particularly in cases where physical distancing may not be feasible (Public Health Agency of Canada, 2020). Emphasis was placed on nonmedical masks serving not to protect the wearer, but rather others who come within close proximity of the wearer. Echoing her public statements on the matter, Canada's chief public health officer Tweeted that “[w]earing a NON-MEDICAL mask in public settings has not been proven to add any protection TO the person wearing it, but it can be an additional way to prevent spread FROM an infected person to others” (Tam, 2020).

## Introduction

The revised guidance on masks from public health officials has been one of the most significant COVID-19 policy reversals to date. Statements made at the outset of the pandemic, including those from the World Health Organization (WHO), the United States Surgeon General, and the Chief Public Health Officer of Canada, all actively discouraged asymptomatic members of the general public from wearing masks. However, on April 3, 2020, the United States Center for Disease Control and Prevention (CDC) issued new recommendations that called for nonmedical masks, such as cloth face coverings, to be worn in public settings where other social distancing measures are difficult to maintain (Adams, 2020). Canadian public health officials quickly followed with their own guidance for wearing nonmedical masks or face coverings when out in public; however, they have stressed that doing so is optional for asymptomatic persons and should be seen as a complement to existing precautionary measures such as physical distancing and hand hygiene, particularly in cases where physical distancing may not be feasible (Public Health Agency of Canada, 2020). Emphasis was placed on nonmedical masks serving not to protect the wearer, but rather others who come within close proximity of the wearer. Echoing her public statements on the matter, Canada's chief public health officer Tweeted that “[w]earing a NON-MEDICAL mask in public settings has not been proven to add any protection TO the person wearing it, but it can be an additional way to prevent spread FROM an infected person to others” (Tam, 2020).

**Figure 1.**
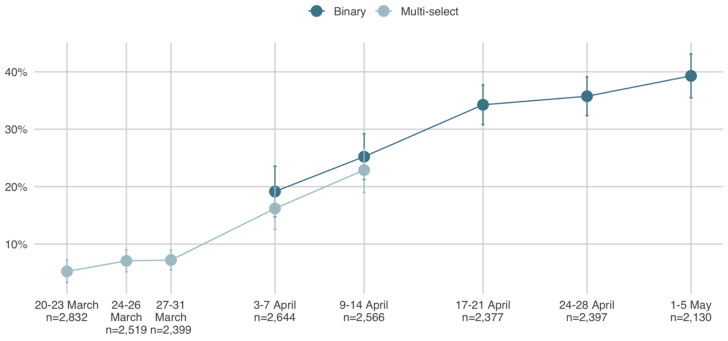
Mask usage in Canada time series

*Source:* Vox Pop Labs COVID-19 Monitor. *Notes:* Dates and sample sizes of each wave are reported along the *x*-axis. Respondents were asked, “What changes, if any, have you made to your normal routine in response to the COVID-19 pandemic?” “Wearing a mask” was among the response options. Respondents to the first three waves of the study were provided with the response options in a multiselect format and asked to select all that applied. Subsequent waves transitioned to a binary scale in which respondents were asked to explicitly respond “Yes” or “No” to each response option. To ensure that the revised format did not affect self-reported behavioural changes, respondents were randomly assigned either the multiselect or the binary scale for the fourth and fifth waves. As the differences in self-reported behaviours were not statistically significant, the binary scale was adopted for all respondents from the sixth wave onward.

Findings from a multiwave study conducted by Vox Pop Labs indicate that the prevalence of mask usage among Canadians rose significantly upon the issuance of revised guidance on the matter from U.S. and Canadian public health officials and has since continued its upward orientation (see Figure 1). Given the discursive framing of wearing masks as a common good rather than an individual benefit, we examine the extent to which the rise in mask usage is motivated by collective interest as opposed to self-interest. Drawing on recent survey data, we find that the decision to wear a mask is in part a function of collective interest. Specifically, the increased propensity among Canadians to wear masks is to a limited extent driven by concern for the welfare of others as opposed to oneself. However, the effect of collective interest on mask usage is rather modest by comparison with regional, gender, and partisan dynamics. We find no evidence to indicate that priming self-interest has an effect on mask usage by individuals in the general population.

## Masks as a Collective Action Problem

Mask usage serves as a useful example of how collective action operates in the context of COVID-19. According to Olson (1965), individuals in a group behave as rational egoists and would thus be disinclined to wear a mask if does not offer them additional protection from personally contracting COVID-19. Even though wearing a mask may indirectly protect the wearer in that increased mask usage by the general public may reduce overall transmission of COVID-19, Olson's logic asserts that individuals would instead free ride based on the expectation that other group members would adopt mask usage. This view is challenged by theorists who argue that rational self-interest alone fails to appropriately capture the empirically observable dynamics of collective action (Mansbridge, 1990). Ostrom (2000, p. 142) argues that a substantial proportion of the population is composed of so-called “conditional cooperators” who are generally willing to act in the collective interest as long as they see a sufficient degree of reciprocation by others. Conditional cooperators would be willing to don a mask to protect others so long as they observe a sufficient number of people within their group doing the same.

## Data and Method

We employed data from the fourth wave of a rolling sample survey, which was fielded between April 3 and April 7, 2020 and completed by 2,194 respondents who currently reside in Canada. The sample was drawn from the Vox Pop Labs online panel (N ~ 650,000) as part of its COVID-19 Monitor initiative, a 24-wave weekly survey on public opinion in relation to the COVID-19 pandemic. The sample was pre-stratified according to age, sex, education, partisanship, and region.

We tested whether Canadians exhibit a higher propensity to wear masks in response to appeals to a sense of collective interest or self-interest. To do so, we designed a survey experiment in which respondents were randomly presented with one of three texts: a control, a collective interest treatment, and a self-interest treatment. The control text read as follows:

Going forward, how likely are you to voluntarily (i.e., without being required to do so) wear a mask or any sort of protective face covering out in public as a preventative measure against COVID-19?

The collective interest treatment included the following preamble prior to the control text:

Some countries have started asking their citizens to cover their faces when in public in order **to avoid potentially transmitting the virus to others with whom they come into contact**. The suggestion is that, **by wearing a mask, you may be protecting others from infection**.

The self-interest treatment included the following preamble prior to the control text:

Some countries have started asking their citizens to cover their faces when in public in order **to avoid potentially contracting the virus from others with whom they come into contact**. The suggestion is that, **by wearing a mask, you may be protecting yourself from infection**.

Survey respondents were asked to indicate their response on an 11-point scale ranging from 0 to 10, where 0 meant “no more likely” and 10 meant “much more likely.” The mean of this continuous variable serves as our outcome variable. We used linear regression to model the effects of each treatment on the likeliness to wear a mask going forward.

## Results

The results of the study are summarized in Table 1. Model 1 compares each of the two treatments (collective interest and self-interest) against the control group. The coefficients represent the respective averages of the control and each of the treatment groups on the 11–point response scale. In Model 2, we include a series of sociodemographic regressors in order to allow for substantive comparison of the effect size of the treatments with those of other independent variables.

**Table 1:** Linear regression results

	*Dependent variable*	
	Likelihood to voluntarily start wearing a mask	
	Model 1	Model 2
**Treatments**		
Self-interest	0.111 (0.171)	0.065 (0.165)
Collective interest	0.505*** (0.170)	0.457*** (0.164)
**Education (high school or below)**		
College		0.373* (0.220)
University		0.305 (0.206)
**Age group (18–24)**		
25–44		−0.105 (0.331)
45–64		0.170 (0.333)
65+		0.242 (0.346)
**Sex (men)**		
Women		0.593*** (0.136)
**Region (AB)**		
ATL		0.351 (0.386)
BC		0.624** (0.293)
MBSK		0.171 (0.371)
ON		0.583** (0.238)
QC		−1.041*** (0.242)
**Vote (BQ)**		
CPC		0.415 (0.278)
GPC		0.447 (0.311)
LPC		0.650*** (0.241)
NDP		0.950*** (0.268)
OTH		0.971*** (0.314)
PPC		0.403 (0.632)
Constant	4.852*** (0.122)	3.808*** (0.454)
Observations	2,194	2,194
R2	0.004	0.089
Adjusted R2	0.004	0.081
Residual std. error	3.251 (df = 2191)	3.212 (df = 2174)
F statistic	4.924*** (df = 2;2191)	11.191*** (df = 19;2174)

*Note:* *p < 0.1; **p < 0.05; ***p < 0.01.

Model 1 indicates that the self-interested treatment is not statistically significant vis-à-vis the baseline control. Suggesting that Canadians should wear masks as a protective measure against contracting COVID-19 does not appear to increase the probability that they will do so. However, we do observe a small but statistically significant effect when it comes to the collective interest treatment. When compared with the control group, the collective interest treatment increases the average respondent's inclination to wear a mask by 0.505 points on the 11–point continuous scale. Though a five-percentage-point increase on an 11-point scale is modest, it is non-negligible. The finding is statistically significant when the control is set as the base category, but also when the self-interested treatment acts as the baseline (see the Appendix).

**Figure 2.**
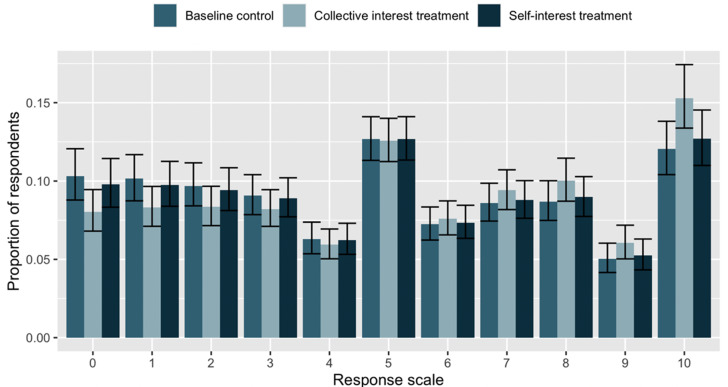
Ordinal regression predicted values.

The results of Model 1 demonstrate that Canadians are more willing to wear masks as a measure to protect others from COVID-19 rather than themselves. Figure 2 displays the results of an ordered logit regression so as to examine the distribution of responses across the 11-point scale for the control group and both of the treatments. We observe that the differences in the collective treatment and self-interest treatments cluster at the ends of the scale, whereas there are similar proportions of respondents in the centre in every case. This suggests that collective interest primers reduce opposition and increase support for wearing masks at the extremes.

Model 2 adds additional categorical independent variables including sex, age group, region, highest level of educational attainment, and vote choice in the 2019 Canadian federal election. The results observed in Model 1 are robust to the inclusion of additional regressors included in Model 2, both in terms of significance and effect size. Though Model 2 also serves in principle to support the argument that individuals can act in the collective interest under certain conditions, it behooves us to note both the significance and effect size of several of the included sociodemographic control variables. First, women are more likely than men to wear masks. The effect size is larger than that of the collective interest treatment. Second, living in British Columbia is associated with a higher likelihood of wearing a mask, while living in Quebec is associated with a substantively lower likelihood of doing so. Compared to the (alphabetically determined) baseline of Alberta, the effect size for Quebec is −1.041, which is the largest effect size of any of the variables included in the model and a difference of 1.665 points from BC on the 11-point response scale. Further study is required to interrogate this difference, but these differences may be related to the mixed and controversial messaging around the use of masks from Premier François Legault and National Director of Public Health Horracio Arruda (Boisvert, 2020; Cardinal, 2020). Third, partisan differences have an effect on the adoption of masks insofar as we observe a substantive and significant effect on mask uptake by those who voted for the Liberal Party and New Democratic Party in the 2019 Canadian federal election. Liberal and NDP supporters are more likely to wear masks, whereas the result for Conservative Party, Green Party, and Bloc Québécois voters is not significant. There is likely an ideological dimension at play within these findings, with left-leaning Canadians being particularly more receptive to the idea of wearing masks.

## Conclusion

Although in substantive terms the effect size is relatively modest, the findings of this study demonstrate that Canadians are significantly more likely to adopt mask-wearing in public when doing so is seen as a means to protect others from COVID-19 rather than as a means to protect themselves. Indeed, at the time of writing, the dominant framing around the utility of wearing masks in public was to prevent the potential transmission of COVID-19, rather than as means to prevent oneself from contracting the virus. The survey results suggest that this approach is more likely to induce compliance with directives to wear masks than either a generic appeal or one that speaks to self-interest.

Although this study focuses exclusively on the adoption of masks, its findings are potentially instructive in terms of framing broader public health advice in relation to COVID-19 in such a manner as to elicit compliance. The findings also lend credence to theories of collective action that are critical of the idea of rational self-interest as the ubiquitous and exclusive motivation of individuals within a group.

## Appendix

Table 1 reports the effect of the self-interested and collective treatment vis-à-vis the control baseline but does not explicitly compare both treatments. As a robustness check, Table A1 contemplates the self-interested treatment as the baseline. The collective interest treatment remains statistically significant.

**Table A1:** Linear regression results with self-interest treatment as the base category

	*Dependent variable:*
	Likelihood to voluntarily start wearing a mask
Collective interest treatment	0.394*** (0.169)
Self-interest treatment (baseline)	4.963*** (0.121)
Observations	1,479
R2	0.004
Adjusted R2	0.003
Residual std. error	3.253 (df = 1477)
F statistic	5.421** (df = 1;1477)

*Note:* *p < 0.1; **p < 0.05; ***p < 0.01.
